# Blood Translation Elongation Factor-1δ Is a Novel Marker for Cadmium Exposure

**DOI:** 10.3390/ijms14035182

**Published:** 2013-03-04

**Authors:** Qian Lu, Yi-Xiong Lei, Chao-Cai He, Zi-Ning Lei

**Affiliations:** School of Public Health, Guangzhou Medical University, Guangzhou 510182, China; E-Mails: lanlan_fu2@hotmail.com (Q.L.); zhuhuan_bj@hotmail.com (C.-C.H.); weimingli_bj@hotmail.com (Z.-N.L.)

**Keywords:** cadmium, translation elongation factor-1δ, biomarker, toxicity

## Abstract

Translation elongation factor-1δ (TEF-1δ) has been identified as a novel cadmium-responsive proto-oncogene. However, it is still unclear whether TEF-1δ could be a potential biomarker of cadmium exposure. Rats were treated with CdCl_2_ at different concentrations (high dose 1.225, mid-dose 0.612 and low dose 0.306 mg/kg body weight, respectively) for 14 weeks, and the cadmium levels, weight coefficients, serum alanine aminotransferase (ALT), aspartate aminotransferase (AST), blood urea nitrogen (BUN), serum creatinine (SCR), 24-h urine protein (24hPro), urinary creatinine (Cr) and pathological features were determined. The TEF-1δ expression in white blood cells and multiple organs were examined by reverse transcription polymerase chain reaction (PCR) and were also confirmed with fluorescence quantitative PCR. A cadmium dose-dependent increase (*p* < 0.05) of cadmium levels in blood, urine, liver, kidney, heart and lung, and the weight coefficients was observed. The liver and renal function indictors including AST, ALT, SCR, BUN and 24hPro, were elevated in a cadmium dose-dependent manner (*p* < 0.05). Significant pathological changes in liver, kidney, heart and lung were indicated. The TEF-1δ expression was up-regulated in both blood and organs (*p* < 0.05). Moreover, the expression level of blood TEF-1δ was positively correlated to TEF-1δ expression level, cadmium level and toxicity in the organs (*p* < 0.01). This study indicates that blood TEF-1δ is a novel valuable biomarker for cadmium exposure and its organ toxicity.

## 1. Introduction

Cadmium and its compounds are considered as a harmful pollutant worldwide [1–3]. Cadmium has a long biological half-time (19–30 years) and can accumulate and be present in multiple organs for a long term. Cadmium is toxic to organs and can lead to a number of diseases including liver and kidney injury, respiratory diseases, neurological disorders, skeletal system damage and reproductive system damage [4–9]. Cadmium is ranked as the 7th priority toxicant according to the Agency for Toxic Substances and Disease Registry (ATSDR) in the United States [10]. Based on epidemiology and laboratory evidences, cadmium has been reported to cause cancer in many organs including lung, kidney, liver, prostate, pancreas, bladder and breast [11–15]. At 1993, cadmium and its compounds were named as Group 1 carcinogen by the International Agency for Research on Cancer (IARC) [16,17]. Therefore, a sensitive and specific biomarker for cadmium exposure is beneficial.

Recently, we have identified a mouse translation elongation factor-1δ sub-unit (TEF-1δ, GenBank Accession Number AF304351) as a novel cadmium-responsive proto-oncogene [18,19]. However, it is still unclear whether the abnormal expression of TEF-1δ can be a novel biomarker for cadmium exposure. Thus, in this study, we investigated the relationship between the expression of TEF-1δ with cadmium accumulation and toxicity in a rat model of sub-chronic cadmium exposure.

## 2. Materials and Methods

### 2.1. Experimental Animal and Cadmium Exposure Proceduces

Specific-pathogen-free (SPF) Sprague-Dawley (SD) rats (90 ± 10 g) were obtained from Guangdong Medical Laboratory Animal Center (Licence No.: SCXK 2008-0002, Guangdong, China) and maintained under pathogen-free conditions in Laboratory Animal Center of Guangzhou Army General Hospital [Licence No.: SYXK (Military) 2007-33, 2008C1230034834, Guangdong, China]. Ninety-six SD rats (half male and half female) were randomly divided into 4 groups. Rats were sub-chronically exposed to cadmium by intra-peritoneal injection 0.5 mL of 0.9% sodium chloride (NaCl, purity 99.5%, Sigma, St. Louis, MO, USA) solution containing cadmium chloride (CdCl_2_, purity 99%, Sigma, St. Louis, MO, USA) at different concentrations (high dose 1.225, mid-dose 0.612 and low dose 0.306 mg/kg body weight, respectively), and rats in the control group were intra-peritoneal injected with 0.5 mL of 0.9% NaCl solution. Cadmium exposure was performed five times every week. After 14 weeks of exposure, 24 h urine samples were collected. At the second day, rats were anesthetized and the blood was collected from the heart and stored at 4 °C. Tissues of the liver, kidney, heart and lung were harvested and kept at liquid nitrogen. For all the animal experiments, animal handling and experimental procedures were approved by the Animal Experimental Ethics Committee of Guangzhou Army General Hospital (Guangzhou, China).

### 2.2. Cadmium Determination and Organ Functional and Pathological Examination

The cadmium level was determined using the cadmium standard solution (BZ/WJ/GB101/2009-1, Guangdong Occupational Health Inspection Center, Guangdong, China) and atomic absorption spectrometry (ZEENIT700, Analytik Jena, Jena, German). The concentration of cadmium in urine was normalized by urinary creatinine (Cr). Tissue samples were fixed with 10% formalin and the pathological features were examined following the standard Hematoxylin and Eosin (HE) staining protocol. Serum alanine aminotransferase (ALT) and aspartate aminotransferase (AST) were used as biochemical markers of liver function. Blood urea nitrogen (BUN), serum creatinine (SCR) and 24-h urine protein (24hPro) were used as renal function biochemical indicators. ALT, AST, BUN, SCR and 24hPro were measured using corresponding kits according to manufacturer’s instructions and automatic biochemistry analyzer (Hitachi 7600- 020/7170A: Tokyo, Japan).

### 2.3. Total RNA Isolation and TEF-1δ Expression Detection

Total RNA in the white blood cells was isolated using QIAamp RNA Blood Mini Kit (Qiagen, Hilden, Germany). Total RNA in tissues was extracted using TRIzol kit (Gibco BRL, USA) according to manufacturer’s instructions. The purity and integrity of total RNA were analyzed by spectrophotometry (Thermo Fisher Scientific, Inc.: Wilmington, DE, USA) and agarose gel electrophoresis, respectively. The expression of TEF-1δ mRNA was detected with reverse transcription-polymerase chain reactions (RT-PCRs) using Reverse Transcription System (Promega: Madison WI, USA), and the RT-PCR products were semi-quantified using 2% argrose gel (TaKaRa: Tokyo, Japan) and Tanon Gel Image System (GIS-2009, Tanon: Shanghai, China). Furthermore, the quantitative detection of the TEF-1δ mRNA was performed with fluorogenic quantitative PCR (FQ-PCR) using real-time PCR master mix kit (Da’an Co.: Guangzhou, China) in the PE 7000 Sequence Detection System (ABI PRISM: Foster City, CA, USA) using β-actin as internal standard. The primers used in RT-PCR and FQ-PCR were listed in Table 1. For the TEF-1δ analysis with FQ-PCR assay, amplification cycle was programmed as: 42 °C for 10 min, 93 °C for 2 min, and 40 cycles of 93 °C for 45 s and 55 °C for 45 s. The standard preparation, negative control, and target samples were all tested in triplicates. For relative quantification, the expression levels of target genes were automatically calculated according to standard gradient template curve.

### 2.4. Statistical Analysis

All the experiments in this study were repeated three times. The experimental data were shown as mean ± standard error (SE) (*χ̄* ± SE). The data is changed into normal distribution with logarithm if the original data is positive skewness distribution. Data is firstly tested for homogeneity of variance. Analysis of variance, Student-Newman-Keulsa, and Pearson’s correlation will be used if the variance is homogenous. Kruskal-Wallis, Games-Howell test, as well as spearman’s correlation analysis will be used if the data is not homogenous. All the analyses were carried out using the SPSS17.0 software (SPSS Inc.: Chicago, IL, USA). Values less than 0.05 were considered to be statistically significant.

## 3. Results

### 3.1. Elevated Weight Coefficient in Cadmium-Exposed Rats

Rats were divided to 4 groups and treated with CdCl_2_ at different concentrations (high dose 1.225, mid-dose 0.612 and low does 0.306 mg/kg body weight, respectively) or with 0.9% NaCl as control. No weight difference was found between the cadmium-exposed rats and control rats, suggesting that cadmium exposure did not affect the growth and the weight of rats. However, a dose-dependent elevation of the weight coefficient (organ/body weight ratio) was observed in the liver, kidney, heart and lung in cadmium-exposed rats compared to control rats (*p* < 0.05) (Table 2), suggesting the toxicity of cadmium to the above organs.

### 3.2. Accumulation of Cadmium in the Blood, Urine and Multiple Organs

The cadmium concentration was increased in the blood, urine, liver, kidney, heart and lung of the cadmium-exposed rats (Tables 3 and 4) compared to the control group (*p* < 0.05). Among the treated rats, a dose dependent increase of cadmium levels was observed (*p* < 0.05). Importantly, the cadmium levels in the liver and kidney were significant higher than that in heart and lung, suggesting that liver and kidney are two major target organs of cadmium toxicity.

### 3.3. Liver and Renal Injury in Cadmium-Exposed Rats

**Figure 1:**
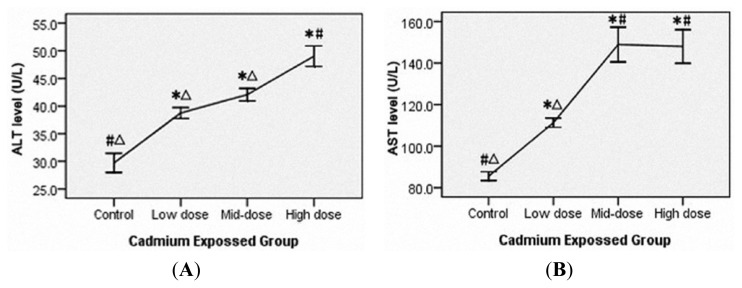

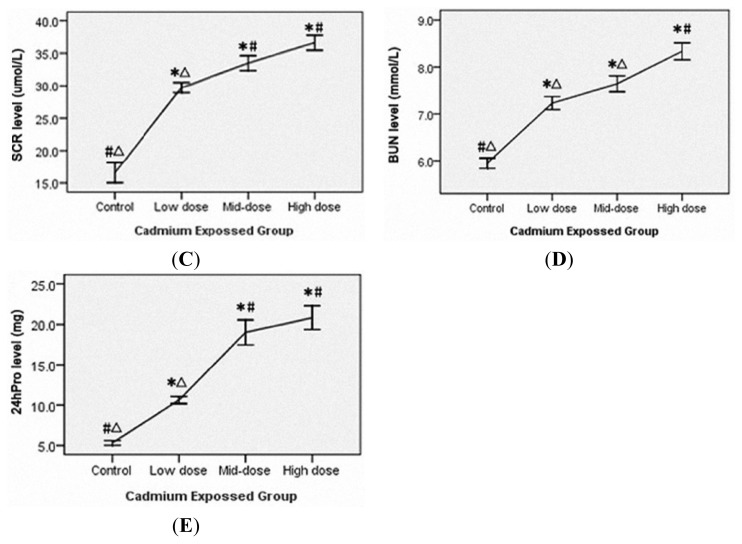
Serum alanine aminotransferase (**A**), aspartate aminotransferase (**B**), serum creatinine (**C**), blood urea nitrogen (**D**) and 24-h urine protein (**E**) levels are elevated in rat exposed to cadmium for 14 weeks. Rats were divided into four groups and treated with CdCl_2_ at three different concentrations (high dose 1.225, mid-dose 0.612 and low does 0.306 mg/kg body weight, respectively) or with 0.9% NaCl as the control. ALT, serum alanine aminotransferase. AST, aspartate aminotransferase. BUN, blood urea nitrogen. SCR, serum creatinine. 24hPro, 24-h urine protein. All the data were shown as *χ̄* ± SE. * means *p* < 0.05 compared to corresponding control group. ^#^ means *p* < 0.05 compared to corresponding low exposure group. ^Δ^ means *p* < 0.05 compared to corresponding high exposure group (Analysis of variance and Student-Newman -Keuls, or Kruskal-Wallis and Games-Howell test).

Serum ALT and AST levels were used as liver function indicators and a dose-dependent elevation was observed in the cadmium-exposed rats (*p* < 0.05) (Figure 1A,B). In addition, BUN, SCR and 24hPro were used as the renal function biochemical indicators and a dose-dependent increase was also observed (*p* < 0.05) (Figure 1C–E).

### 3.4. Pathological Changes of Organ Toxicity from Cadmium Exposure

**Figure 2:**
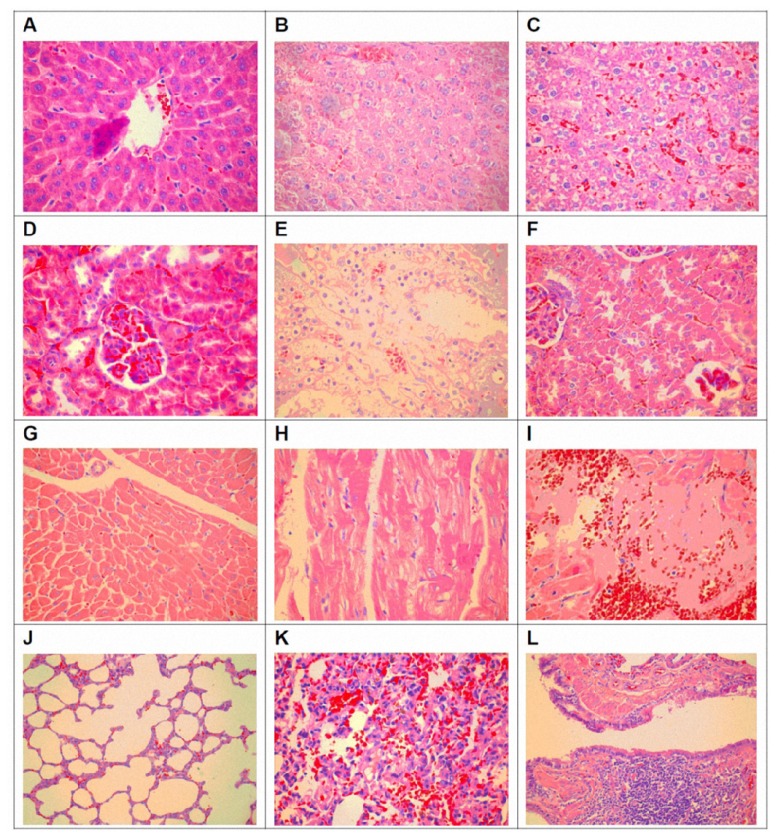
Representative pathological changes (HE staining, ×400) of liver (**A–C**), kidney (**D–F**), heart (**G–I**) and lung (**J–L**) in rats from the control group (**A**,**D**,**G**,**J**), the low cadmium exposure group (B,E,H,K) and the high cadmium exposure group (**C**,**F**,**I**,**L**).

The histological features of liver, kidney, heart and lung in cadmium exposed rats were examined and obvious pathological changes were found in all the organs at different levels, and the changed features were much more obvious in rats exposed with higher cadmium concentration (Figure 2A–C in liver, D–F in kidney, G–I in heart and J–L in lung). Liver damages included indistinct lobular architecture, disappeared liver cord, large areas of hemorrhage, extensive liver cell degeneration such as balloon-like degeneration, liver cell shrinkage, fragmentation and cell disintegration, fusion cells and inflammatory cell infiltration (Figure 2A–C). Renal damages included tubular (mainly proximal tubule) cell swelling, vacuolar degeneration, nuclear pyknosis, necrosis and luminal atrophy interstitial inflammatory infiltration (Figure 2E). Renal tubular cell swelling, disappeared epithelial cell structure, necrosis, exfoliation to the lumen blockage, a small canal stenosis, glomerular structural disorder, red blood cells outside the plaza, and a wide range of renal interstitial congestion were identified (Figure 2F). Heart tissue damage included cloudy swelling of myocardial fibers, nuclear condensation, fragmentation, cell disintegration, stripes disappear, the gap widened, uneven coloring, interstitial congestion, with scattered necrotic foci (Figure 2H,I). Lung tissue damage included pulmonary interstitial widening, interstitial fibrosis, alveolar septal rupture, rupture of alveolar fusion, a large number of alveolar collapse and the degree of heterogeneity, interstitial lung congestion, showing interstitial inflammatory cell infiltration in the lung (Figure 2K). Swelling of the tracheal mucosa, columnar epithelial degeneration, loss of incomplete removal, and a large number of lymphocyte infiltration were found (Figure 2L). In addition, no obvious difference was found between male rats and female rats.

### 3.5. TEF-1δ Is Elevated in Cadmium-Exposed Rats

**Figure 3:**
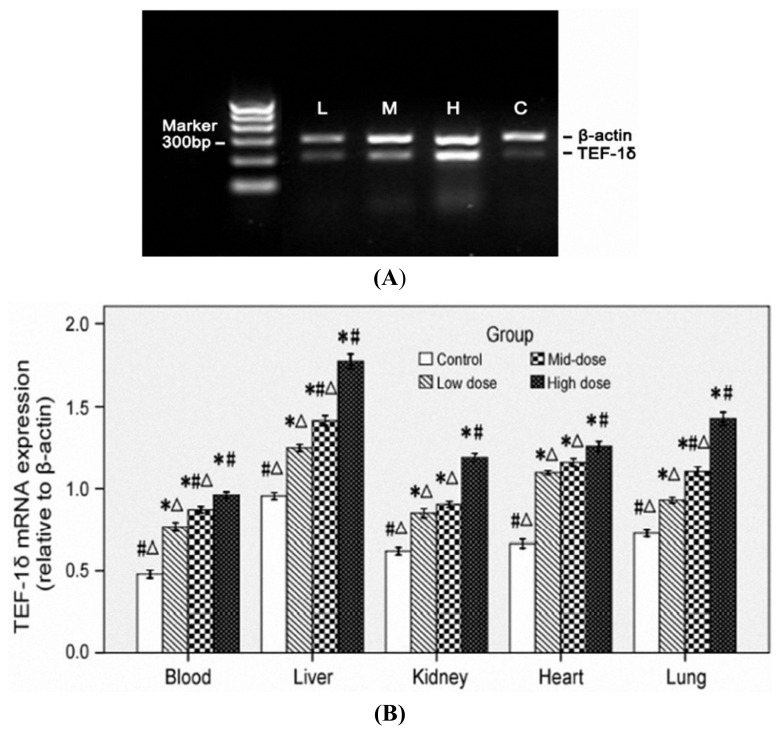
Representative results (**A**) and semi-quantitative analysis (**B**) of TEF-1δ mRNA expression in the blood and major organs of cadmium-exposed rats using RT-PCR assay.

A dose-dependent increase of the TEF-1δ mRNA expression levels was indicated in blood and all the measured organs (liver, kidney, heart and lung) in all cadmium exposed groups (*p* < 0.05) (Figure 3A,B). The increased expression of TEF-1δ in cadmium-exposed rats was further confirmed by FQ-PCR. As shown in Table 5, significant up-regulated TEF-1δ mRNA levels were found in all the detected organs in cadmium-exposed rats. Additionally, an obvious cadmium dose-dependent increase of the TEF-1δ expression level was observed in all tested organs among different cadmium-exposed rats (*p* < 0.05).

### 3.6. TEF-1δ Expression Is Correlated with Cadmium Exposure and Toxicity

**Figure 4:**
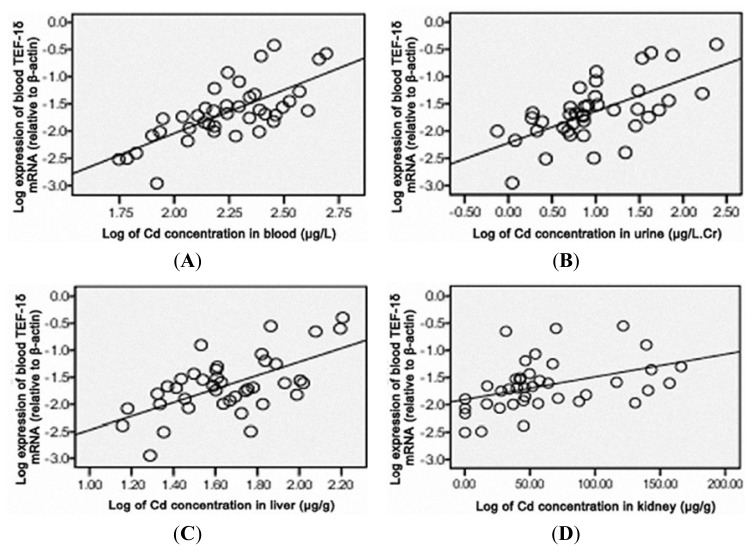

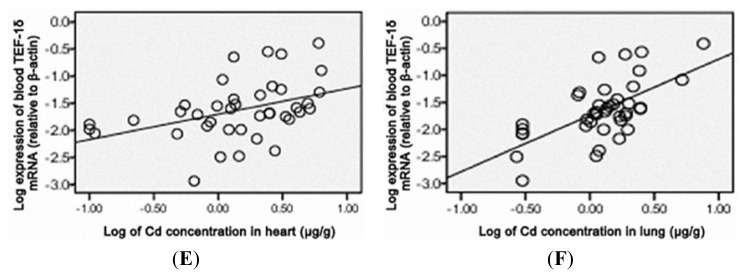
Correlation analysis between the blood TEF-1δ expression and cadmium levels in blood (**A**), urine (**B**), liver (**C**), kidney (**D**), heart (**E**) and lung (**F**) from cadmium-exposed rats. Blood TEF-1δ mRNA expression was calculated by the ratio of its expression to that of β-actin. The urinary cadmium level was normalized by creatinine (μg/L.Cr). The data were changed into normal distribution with logarithm, and the linear relationship was analyzed by Pearson or spearman’s correlation. Blood TEF-1δ expression level showed positively correlation to blood cadmium (r = 0.711, *p* < 0.01), urine cadmium (*r* = 0.631, *p* < 0.01), liver cadmium (*r* = 0.610, *p* < 0.01), renal cadmium (*r* = 0.598, *p* < 0.01), heart cadmium (*r* = 0.527, *p* < 0.01) and lung cadmium (*r* = 0.633, *p* < 0.01).

**Figure 5:**
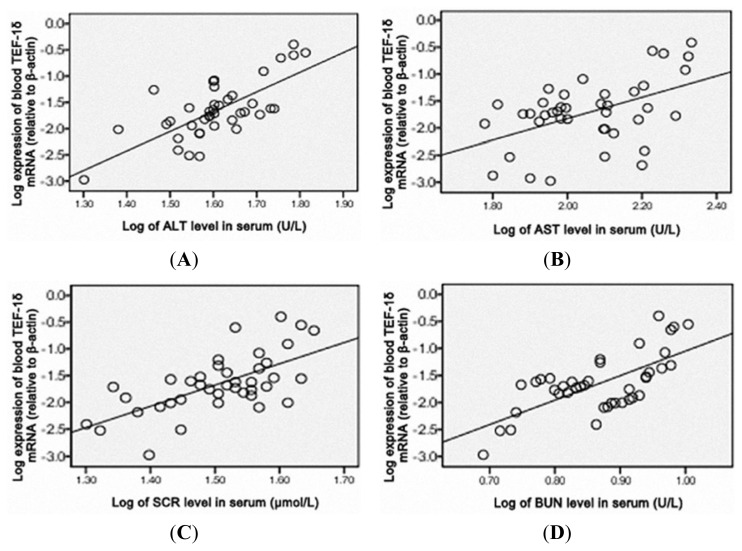

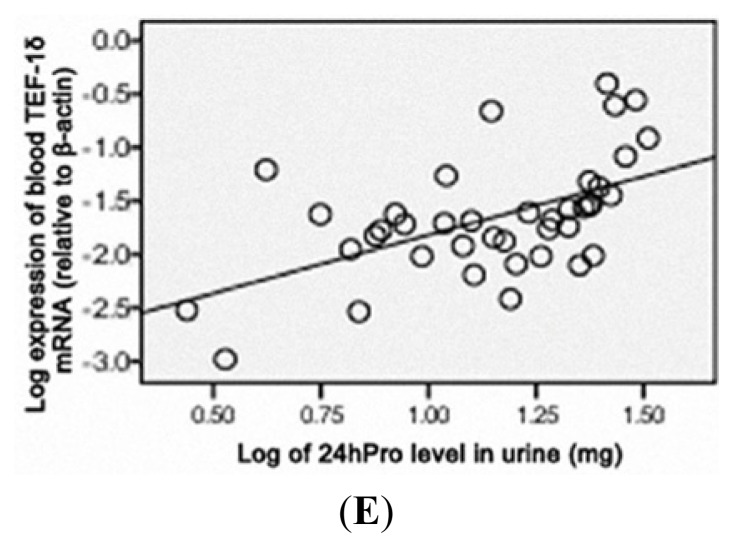
Correlation analysis between the blood TEF-1δ expression and ALT (**A**), AST (**B**), SCR (**C**), BUN (**D**) and 24hPro (**E**) levels in cadmium-exposed rats. Blood TEF-1δ expression level showed positively correlation to ALT (r = 0.663, *P* < 0.01), AST (r = 0.590, *P* < 0.01), SCR (r = 0.648, *P* < 0.01), BUN (r = 0.583, *P* < 0.01) and 24hPro (r = 0.543, *P* < 0.01). ALT, serum alanine aminotransferase. AST, aspartate aminotransferase. BUN, blood urea nitrogen. SCR, serum creatinine. 24hPro, 24-h urine protein.

**Figure 6:**
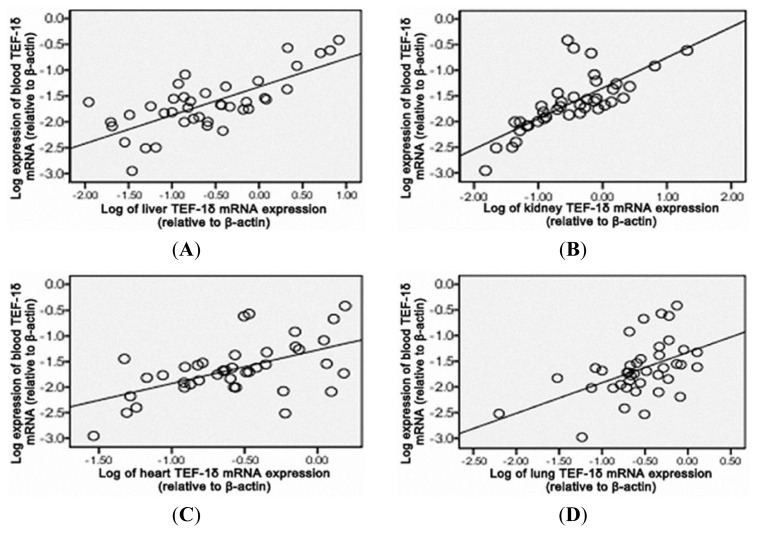
Pearson or spearman’s correlation analysis of TEF-1δ expression levels between the blood and liver (**A**), kidney (**B**), heart (**C**) and lung (**D**) in cadmium-exposed rats. Blood TEF-1δ expression level showed positively correlation to the TEF-1δ levels in liver (*r* = 0.639, *p* < 0.01), kidney (*r* = 0.609, *p* < 0.01), heart (*r* = 0.651, *p* < 0.01) and lung (*r* = 0.628, *p* < 0.01).

As shown in Figure 4, a positive correlation of the blood TEF-1δ expression level was observed with the cadmium levels in blood, urine, liver, kidney, heart and lung (*p* < 0.01), liver function indicators (ALT and AST) and renal function indicators (SCR, BUN and 24hPro) (*p* < 0.01) (Figures 5 and 6). All these results strongly indicated that the blood TEF-1δ level can reflect the cadmium level in the blood, urine and other major organs, as well as the toxicities to these organs.

## 4. Discussion

Many cellular and molecular events are involved in the toxic effects of chemical carcinogens in the body [20,21]. It is well recognized that abnormal expression of eukaryotic translation factors facilitates the malignant transformation of primary cells and promotes carcinogenesis [22–24]. Accumulating evidences have demonstrated the important roles of the over-expression of translation factors in many kinds of cancers from pancreas, colon, breast, lung and prostate [25–29]. Our previous study has identified a novel mouse cadmium-responsive proto-oncogene TEF-1δ [18,19]. Recently, the abnormal TEF-1δ expression was reported to be involved in the cadmium-induced malignant transformation of human bronchial epithelial cells [20]. The present study further demonstrated that TEF-1δ could be a potential biomarker for cadmium exposure.

The animal model of sub-chronic cadmium exposure we used in this study was developed by continuous intra-peritoneal injection of the CdCl_2_ solution for 14 weeks. The evaluation of the cadmium toxicity included weight coefficient, histo-pathological examination and liver and renal function indicators (ALT, AST, SCR, BUN and 24hPro) analysis. The accumulation of heavy metal is a widely used indicator of its toxicity. The metal levels in blood reflect recent exposures, and the levels of metal in urine reflect body burden from longer term exposure, while levels in tissues reflect the metal accumulation and organ damage [30–32]. This study found a significant accumulation of cadmium in blood, urine and major organs (liver, kidney, heart and lung) in cadmium-exposed rats. The cadmium levels in kidney and liver were much higher than that in heart and lung, suggesting that the former organs are the most major target organs of cadmium, which is consistent with the epidemiological observation [33–36]. Weight coefficient (organ/body weight ratio) is a sensitive, effective and economic indicator of the toxicology, and it is also important in the identification of target organ of toxicant [37,38]. Here we found that the weight coefficients of liver, kidney, heart and lung in cadmium-exposed rats were significant higher than that in control rats, further supporting the toxicity of cadmium to these organs.

Some carcinogens induce organ toxicity by regulating enzyme activities. ALT and AST are sensitive indicators of liver damage [37,39]. Our study found that the ALT and AST levels were significant increased after cadmium-exposure, indicating that cadmium could induce serious liver damage, which was further confirmed by histo-pathological examination. Previous work has showed that kidney was one of the major target organs of cadmium [40–42]. In the kidney from cadmium-exposed rats, we found renal proximal tubule cell swelling, vacuolar degeneration, nuclear psychosis, necrosis and atrophy lumen, as well as the elevation of SCR, BUN and 24hPro, suggesting that cadmium induced heavy renal injury. Additionally, we observed a significant pathological change in lung and heart. Lung damages include septal thickening, inflammatory cell infiltration, fibrosis, alveolar septal rupture, alveolar rupture and fusion, alveolar collapse, interstitial lung congestion and airway columnar epithelial degeneration. Heart damages include muscle fibers cloudy swelling, nuclear condensation and fragmentation, cell disintegration, stripes disappear, widened gap, interstitial congestion and scattered necrosis. Interestingly, less has been previously reported regarding the heart damage in environmental cadmium exposure. Our data has identified that the heart might also be an important target organ of cadmium toxicity.

Translation elongation factors (TEFs) constitute a group of nucleotide exchange proteins that are required for the elongation step of translation. TEFs also have non-canonical functions unrelated to protein synthesis [43]. Increasing evidence has shown that translation factors participated in the control of cell proliferation, suggesting that these factors may serve as therapeutic targets for cancer [44,45]. It has been shown that increased expression of TEF1A1 and TEF1A2 proteins were associated with increased cell proliferation, oncogenic transformation, and delayed cell senescence [46,47]. It is well established that cadmium exerts multiple toxicities by affecting cell proliferation, differentiation, apoptosis and other cellular activities through modulation of gene expression by activation of transcription and translation factors [30,48–50]. Our previous work also demonstrated that the DNA repair genes’ epigenetic mechanisms responsible for carcinogens are due to cadmium [51]. In the present study, the blood level of TEF-1δ in cadmium-induced rats showed very well positive correlation to the cadmium levels in major organs and the damage degree of these organs, suggesting that blood TEF-1δ can reflect the accumulation of cadmium in major organs and the organ damage. TEF-1δ expression level in blood is useful in predicting the cadmium-induced toxicity. Taken together, our study firstly identified that blood TEF-1δ is a novel valuable biomarker of cadmium exposure and organ toxicity of cadmium. It may be a significant biomarker for field investigations and risk assessment for humans, which are exposed to occupational and environmental cadmium.

## Figures and Tables

**Table 1:** Sequences of primers for TEF-1δ used in RT-PCR and FQ-PCR detection.

Primer	Sequence	Length
**RT-PCR**		
Forward	5′-ACC AGC AGA GGA CGA TGA-3′	227 bp
Reverse	5′-AAT TGG ATG GAA CGC ACA-3′	
**FQ-PCR**		
Forward	5′-GGAGCGGCTACGGCAGTAC-3′	90 bp
Reverse	5′-TCCCAAGGCTTGACATCCA-3′	
Probe	5′-CACTGGTGGCCAAGTCCTCCATCCTAMARA-3′	

**Table 2:** Examination of weight coefficients (organ/body weight ratios).

Groups	Liver (%)	Kidney (%)	Heart (%)	Lung (%)
High dose	5.368 ± 0.203 *,#	0.921 ± 0.023 *,#	0.428 ± 0.015 *	0.625 ± 0.018 *,#
Mid-dose	4.721 ± 0.181 *	0.898 ± 0.028 *	0.414 ± 0.012 *	0.576 ± 0.014 *
Low dose	4.542 ± 0.154 *,Δ	0.828 ± 0.020 *,Δ	0.397 ± 0.011 *	0.529 ± 0.016 *,Δ
Control	3.950 ± 0.127 #,Δ	0.706 ± 0.015 #,Δ	0.349 ± 0.008 #,Δ	0.457 ± 0.014 #,Δ

All the data were shown as *χ̄* ± SE.

* *p* < 0.05 compared to corresponding control group;

# *P* < 0.05 compared to corresponding low exposure group.

Δ *p* < 0.05 compared to corresponding high exposure group.

**Table 3:** Examination of cadmium levels in blood and urine.

Groups	Blood (μg/L)	Urine (μg/L.Cr)
High dose	313.25 ± 28.81 *,#	38.31 ± 3.78 *,#
Mid-dose	210.72 ± 11.64 *,#,Δ	17.95 ± 1.50 *,#,Δ
Low dose	128.63 ± 8.71 *,Δ	6.49 ± 0.53 *,Δ
Control	0.50 ± 0.04 #,Δ	0.65 ± 0.06 #,Δ

All the data were shown as *χ̄* ± SE.

* *P* < 0.05 compared to corresponding control group.

# *P* < 0.05 compared to corresponding low exposure group.

Δ *P* < 0.05 compared to corresponding high exposure group.

**Table 4:** Examination of cadmium levels in organs.

Groups	Liver (μg/g)	Kidney (μg/g)	Heart (μg/g)	Lung (μg/g)
High dose	133.79 ± 44.36 *,#	141.34 ± 30.87 *,#	5.51 ± 1.66 *,#	3.52 ± 1.48 *,#
Mid-dose	66.99 ± 30.26 *,#,Δ	70.02 ± 16.12 *,#,Δ	2.30 ± 0.87 *,#Δ	1.94 ± 0.42 *,#,Δ
Low dose	39.15 ± 16.95 *,Δ	42.29 ± 14.03 *,Δ	1.02 ± 0.31 *,Δ	1.36 ± 0.54 *,Δ
Control	0.04 ± 0.00 #,Δ	0.22 ± 0.02 #,Δ	0.11 ± 0.02 #,Δ	0.20 ± 0.02 #,Δ

All the data were shown as *χ̄* ± SE.

* *p* < 0.05 compared to corresponding control group.

# *P* < 0.05 compared to corresponding low exposure group.

Δ *p* < 0.05 compared to corresponding high exposure group.

**Table 5:** Quantitative analysis of TEF-1δ mRNA expression (relative to β-actin) in cadmium-exposed rats by FQ-PCR detection.

Groups	Blood	Liver	Kidney	Heart	Lung
High dose	152.87 ± 121.87 *,#	3.00 ± 2.70 *,#	1.27 ± 0.70 *,#	1.00 ± 0.44 *,#	0.82 ± 0.27 *,#
Mid-dose	27.80 ± 7.283 *,#,Δ	0.50 ± 0.31 *,#,Δ	0.43 ± 0.23 *,#,Δ	0.40 ± 0.18 *,Δ	0.40 ± 0.13 *,#,Δ
Low dose	16.50 ± 3.393 *,Δ	0.14 ± 0.03 *,Δ	0.13 ± 0.08 *,Δ	0.24 ± 0.08 *,Δ	0.24 ± 0.05 *,Δ
Control	6.49 ± 3.357 #,Δ	0.04 ± 0.02 #,Δ	0.01 ± 0.01 #,Δ	0.08 ± 0.04 #,Δ	0.10 ± 0.06 #,Δ

Rats were divided to 4 groups and treated with CdCl_2_ at different concentrations (high dose 1.225, mid-dose 0.612 and low does 0.306 mg/kg body weight, respectively) or with 0.9% NaCl as control. The quantitative analysis of TEF-1δ mRNA expression in cadmium-exposed rats was detected by fluorogenic quantitative PCR (FQ-PCR) and the mRNA expression levels were calculated relative to β-actin. All the data were shown as *χ̄* ± SE.

* , *p* < 0.05 compared to corresponding control group.

# *p* < 0.05 compared to corresponding low exposure group.

Δ *p* < 0.05 compared to corresponding high exposure group. (Analysis of variance and Student-Newman -Keuls, or Kruskal-Wallis and Games-Howell test).

## References

[1] Diao W.P., Ni W.Z., Ma H.Y., Yang X.E. (2005). Cadmium pollution in paddy soil as affected by different rice (Oryza sativa L.) cultivars. Bull. Environ. Contam. Toxicol.

[2] Swaddiwudhipong W., Mahasakpan P., Funkhiew T., Limpatanachote P. (2010). Changes in cadmium exposure among persons living in cadmium-contaminated areas in northwestern Thailand: A five-year follow-up. J. Med. Assoc. Thai.

[3] Thun M.J., Elinder C.G., Friberg L. (1991). Scientific basis for an occupational standard for cadmium. Am. J. Ind. Med.

[4] Boguszewska A., Pasternak K. (2004). Cadmium-influence on biochemical processes of the human organism. Ann. Univ. Mariae. Curie. Sklodowska.

[5] Koyu A., Gokcimen A., Ozguner F., Bayram D.S., Kocak A. (2006). Evaluation of the effects of cadmium on rat liver. Mol. Cell Biochem.

[6] Nordberg G., Jin T., Bernard A., Fierens S., Buchet J.P., Ye T., Kong Q., Wang H. (2002). Low bone density and renal dysfunction following environmental cadmium exposure in China. Ambio.

[7] Satarug S., Garrett S.H., Sens M.A., Sens D.A. (2010). Cadmium, environmental exposure, and health outcomes. Environ. Health Perspect.

[8] Schöpfer J., Drasch G., Schrauzer G.N. (2010). Selenium and cadmium levels and ratios in prostates, livers, and kidneys of nonsmokers and smokers. Biol. Trace Elem. Res.

[9] Wennberg M., Lundh T., Bergdahl I.A., Hallmans G., Jansson J.H., Stegmayr B., Custodio H.M., Skerfving S. (2006). Time trends in burdens of cadmium, lead, and mercury in the population of northern Sweden. Environ. Res.

[10] Fay R.M., Mumtaz M.M. (1996). Development of a priority list of chemical mixture occurring at 1188 hazardous waste sites, using the HazDat database. Food Chem. Toxicol.

[11] Benbrahim-Tallaa L., Tokar E.J., Diwan B.A., Dill A.L., Coppin J.F., Waalkes M.P. (2009). Cadmium malignantly transforms normal human breast epithelial cells into a basal-like phenotype. Environ. Health Perspect.

[12] Goyer R.A., Liu J., Waalkes M.P. (2004). Cadmium and cancer of prostate and testis. Biometals.

[13] Kriegel A.M., Soliman A.S., Zhang Q., El-Ghawalby N., Ezzat F., Soultan A., Abdel-Wahab M., Fathy O., Ebidi G., Bassiouni N. (2006). Serum cadmium levels in pancreatic cancer patients from the East Nile Delta region of Egypt. Environ. Health Perspect.

[14] Sorahan T., Esmen N.A. (2004). Lung cancer mortality in UK nickel-cadmium battery workers, 1947–2000. Occup. Environ. Med.

[15] Waalkes M.P. (2003). Cadmium carcinogenesis. Mutat. Res.

[16] Huff J., Lunn R.M., Waalkes M.P., Tomatis L., Infante P.F. (2007). Cadmium-induced cancers in animals and in humans. Int. J. Occup. Environ. Health.

[17] The International Agency for Research on Cancer (IARC) (1993). Beryllium, Cadmium, Mercury and Exposures in the Glass Manufacturing Industry.

[18] Joseph P., Lei Y.X., Whong W.Z., Ong T.M. (2002). Oncogenic potential of mouse translation elongation factor-1 delta, a novel cadmium-responsive proto-oncogene. J. Biol. Chem.

[19] Lei Y.X., Chen J.K., Wu Z.L. (2002). Blocking the translation elongation factor-1δ with its antisense mRNA results in a significant reversal of its oncogenic potential. Teratog. Carcinog. Mutagen.

[20] Thomas R.S., Bao W., Chu T.M., Bessarabova M., Nikolskaya T., Nikolsky Y., Andersen M.E., Wolfinger R.D. (2009). Use of short-term transcriptional profiles to assess the long-term cancer-related safety of environmental and industrial chemicals. Toxicol. Sci.

[21] Vogelstein B., Kinzler R.W. (1993). The mulistep nature of cancer. Trends Genet.

[22] Lee T., Pelletier J. (2012). Eukaryotic initiation factor 4F: A vulnerability of tumor cells. Future Med. Chem.

[23] Riis B., Rattan S.I., Clark B.F., Merrick W.C. (1990). Eukaryotic translation elongation factors. Trends Biochem. Sci.

[24] Sonenberg N. (1993). Translation factors as effectors of cell growth and tumorigenesis. Curr. Opin. Cell Biol.

[25] Chi K., Jones D.V., Frazier M.L. (1992). Expression of an elongation factor 1 gamma-related sequence in adenocarcinomas of the colon. Gastroenterology.

[26] Grant A.G., Flomen R.M., Tizard M.L., Grant D.A. (1992). Differential screening of human pancreatic adenocarcinoma lambda gt11 expression library has identified increased transcription of elongation factor EF-1 alpha in tumor cells. Int. J. Cancer.

[27] Khoury T., Alrawi S., Ramnath N., Li Q., Grimm M., Black J., Tan D. (2009). Eukaryotic initiation factor-4E and cyclin D1 expression associated with patient survival in lung cancer. Clin. Lung Cancer.

[28] Siebke C., James T.C., Cummins R., O’Grady T., Kay E., Bond U. (2012). Phage display biopanning identifies the translation initiation and elongation factors (IF1α-3 and eIF-3) as components of Hsp70-peptide complexes in breast tumour cells. Cell Stress Chaperones.

[29] Yoshizawa A., Fukuoka J., Shimizu S., Shilo K., Franks T.J., Hewitt S.M., Fujii T., Cordon-Cardo C., Jen J., Travis W.D. (2010). Overexpression of phospho-eIF4E is associated with survival through AKT pathway in non-small cell lung cancer. Clin. Cancer Res.

[30] Lei Y.X., Wang M., Wei L., Li M., Lin H.Z. (2010). Alterative expression and sequence analysis of human elongation factor-1δ during malignant transformation of human bronchial epithelial cells induced by cadmium chloride. Biomed. Environ. Sci.

[31] Ikeda M., Ohashi F., Fukui Y., Sakuragi S., Moriguchi J. (2011). Closer correlation of cadmium in urine than that of cadmium in blood with tubular dysfunction markers in urine among general women populations in Japan. Int. Arch. Occup. Environ. Health.

[32] Järup L., Akesson A. (2009). Current status of cadmium as an environmental health problem. Toxicol. Appl. Pharmacol.

[33] Fowler B.A. (2009). Monitoring of human populations for early markers of cadmium toxicity: A review. Toxicol. Appl. Pharmacol.

[34] Menke A., Muntner P., Silbergeld E.K., Platz E.A., Guallar E. (2009). Cadmium levels in urine and mortality among U.S. adults. Environ. Health Perspect.

[35] Nawrot T.S., Staessen J.A., Roels H.A., Munters E., Cuypers A., Richart T., Ruttens A., Smeets K., Clijsters H., Vangronsveld J. (2010). Cadmium exposure in the population: From health risks to strategies of prevention. Biometals.

[36] Peters J.L., Perlstein T.S., Perry M.J., McNeely E., Weuve J. (2010). Cadmium exposure in association with history of stroke and heart failure. Environ. Res.

[37] Asagba S.O., Adaikpoh M.A., Kadiri H., Obi F.O. (2007). Influence of aqueous extract of Hibiscus sabdariffa L. petal on cadmium toxicity in rats. Biol. Trace. Elem. Res.

[38] Bernard A., Lauwerys R, Foulkes E.C. (1986). Effects of Cadmium Exposure in Humans. Handbook of Experimental Pharmacology.

[39] Brzóska M.M., Moniuszko-Jakoniuk J., Piłat-Marcinkiewicz B., Sawicki B. (2003). Liver and kidney function and histology in rats exposed to cadmium and ethanol. Alcohol Alcohol.

[40] Nogawa K., Kobayashi E., Honda R., Ishizaki A., Kawano S., Matsuda H. (1980). Renal dysfunctions of inhabitants in a cadmium-polluted area. Environ. Res.

[41] Tanimoto A., Hamada T., Koide O. (1993). Cell death and regeneration of renaproximal tubular cells in rats with subchronic cadmium intoxication. Toxicol. Pathol.

[42] Zhou Y., Vaidya V.S., Brown R.P., Zhang J., Rosenzweig B.A., Thompson K.L., Miller T.J., Bonventre J.V., Goering P.L. (2008). Comparison of kidney injury molecule-1 and other nephrotoxicity biomarkers in urine and kidney following acute exposure to gentamicin, mercury, and chromium. Toxicol. Sci.

[43] Lamberti A., Caraglia M., Longo O., Marra M., Abbruzzese A., Arcari P. (2004). The translation elongation factor 1A in tumorigenesis, signal transduction and apoptosis. Amino Acids.

[44] Caraglia M., Budillon A., Vitale G., Lupoli G., Tagliaferro P., Abruzzese A. (2000). Modulation of molecular mechanisms involved in protein synthesis machinery as a new tool for control of cell proliferation. Eur. J. Biochem.

[45] Silvera D., Formenti S.C., Schneider R.J. (2010). Translational control in cancer. Nat. Rev. Cancer.

[46] Anand N., Murthy S., Amann G., Wernick M., Porter L.A., Cukier I.H., Collins C., Gray J.W., Diebold J., Demetrick D.J., Lee J.M. (2002). Protein elongation factor EEF1A2 is a putative oncogene in ovarian cancer. Nat. Genet.

[47] Edmonds B.T., Wyckoff J., Yeung Y.G., Wang Y., Stanley E.R., Jones J., Segall J., Condeelis J. (1996). Elongation factor-1 alpha is an overexpressed actin binding protein in metastatic rat mammary adenocarcinoma. J. Cell Sci.

[48] Joseph P., Lei Y.X., Ong T.M. (2004). Up-regulation of expression of translation factors—A novel molecular mechanism for cadmium carcinogenesis. Mol. Cell Biochem.

[49] Kawata K., Shimazaki R., Okabe S. (2009). Comparison of gene expression profiles in HepG2 cells exposed to arsenic, cadmium, nickel, and three model carcinogens for investigating the mechanisms of metal carcinogenesis. Environ. Mol. Mutagen.

[50] Waisberg M., Joseph P., Hale B., Beyersmann D. (2003). Molecular and cellular mechanisms of cadmium carcinogenesis. Toxicology.

[51] Zhou Z.H., Lei Y.X., Wang C.X. Analysis of aberrant methylation in DNA repair genes during malignant transformation of human bronchial epithelial cells induced by cadmium. Toxicol. Sci..

